# Sex Differences in Outcomes of Chimeric Antigen Receptor (CAR) T‐Cell Therapy

**DOI:** 10.1002/cam4.70831

**Published:** 2025-03-25

**Authors:** Jia Yi Tan, Yong Hao Yeo, Hermon Wong Kha Kin, Qi Xuan Ang, Mohammad Muhsin Chisti, Daniel Ezekwudo, Talal Hilal

**Affiliations:** ^1^ Department of Internal Medicine New York Medical College at Saint Michael's Medical Center Newark New Jersey USA; ^2^ Department of Internal Medicine/Pediatrics Corewell Health Royal Oak Michigan USA; ^3^ Universiti Malaysia Sabah Kota Kinabalu Malaysia; ^4^ Department of Internal Medicine Sparrow Health System and Michigan State University East Lansing Michigan USA; ^5^ Hematology and Oncology Corewell Health William Beaumont University Hospital Royal Oak Michigan USA; ^6^ Division of Hematology and Medical Oncology Mayo Clinic Phoenix Arizona USA

**Keywords:** clinical cancer research, hematological cancer, multiple myeloma, non‐Hodgkin's lymphoma

## Abstract

**Background:**

Chimeric Antigen Receptor (CAR) T‐cell therapy has arisen as a revolutionary treatment for hematologic malignancies. Our study aimed to evaluate how sex differences affect outcomes and complications following CAR T‐cell therapy.

**Methods:**

Utilizing the Nationwide Readmissions Database (2018–2020), we identified patients and divided them into male and female groups. Hospital outcomes and complications were compared among these two groups after propensity score matching to match groups based on comorbidities, producing two comparable cohorts.

**Results:**

We analyzed 2928 patients (1832 males, 62.6%, mean age 60.3 ± 13.7 years; 1096 females, 37.4%, mean age 59.1 ± 13.8 years). After propensity score matching (1:1ratio), 1092 males and females were compared. There were no significant sex differences in early mortality (adjusted odd ratios (aOR): 1.04 [95% CI 0.69–1.57]), 30‐day readmissions (aOR: 1.05 [95% CI 0.86–1.30]), or nonhome discharge (aOR: 0.89 [95% CI 0.60–1.31]). Females had higher odds of leukopenia (aOR: 1.26 [95% CI 1.06–1.50]) but lower odds of acute kidney injury (aOR: 0.68 [95% CI 0.52–0.88]).

**Conclusions:**

No sex differences were found in hospital outcomes, including early mortality, 30‐day readmission, and nonhome discharge after CAR T‐cell therapy.

Chimeric Antigen Receptor (CAR) T‐cell therapy has arisen as a revolutionary treatment for hematologic malignancies. Despite its transformative potential, there remains a significant lack of large‐population data addressing how sex influences patient outcomes following treatment. The biological and physiological differences between male and female patients and differences—could potentially affect treatment responses and overall safety. While the ZUMA‐1 trial did not specifically evaluate sex‐based differences [1], JULIET and TRANSCEND trials consistently demonstrated no significant association between patient sex and treatment efficacy or survival endpoints [2, 3]. Recognizing this gap in the literature, we performed a nationwide retrospective study to evaluate gender differences in hospital outcomes and adverse events following CAR T‐cell therapy.

Utilizing the Nationwide Readmissions Database (NRD), patients aged ≥ 18 who received CAR T‐cell therapy from 2018 to 2020 were included and divided them into male and female groups. Hospital outcomes and complications were compared among these two groups. We conducted propensity score matching (caliper of 0.2, 1:1 ratio) to match male and female groups based on comorbidities, producing two comparable cohorts. This approach minimizes potential confounding factors, particularly gender‐related differences in comorbidities, thereby increasing the validity of our findings. We conducted further analyses using propensity score‐matched study populations. We first performed univariable matched analysis, then included patients' variables in a multivariable model for conditional logistic regression analyses. We used R studio software for analyses.

A total of 2928 patients admitted for CAR T‐cell therapy during the study period were identified, including 1832 males (62.6%, average age 60.3 ± 13.7 years) and 1096 females (37.4%, average age 59.1 ± 13.8 years). Among male patients, 75.1% were diagnosed with non‐Hodgkin lymphoma (NHL), 12.2% with multiple myeloma (MM), 3.6% with acute lymphocytic leukemia (ALL), and 9.1% with unspecified malignancy. Among female patients, 70.9% were diagnosed with NHL, 13.2% with MM, 5.1% with ALL, and 10.8% with unspecified malignancy. After propensity score matching, there were 1092 males and 1092 females (Table 1). Throughout the study period, there were no significant changes in postprocedural early mortality rates (mortality within 30 days) in both sexes (males, 1.9% in 2018 Q1‐Q2 to 5.7% 2020 Q3‐Q4 [*p* = 0.47]; females, 5.4% in 2018 Q1‐Q2 to 6.9% 2020 Q3‐Q4 [*p* = 1.00]); there were also no significant changes in postprocedural 30‐day readmission rates in both sexes (males, 23.1% in 2018 Q1‐Q2 to 17.6% 2020 Q3‐Q4 [*p* = 0.06]; females, 20.8% in 2018 Q1‐Q2 to 19.3% 2020 Q3‐Q4 [*p* = 0.45]).

**TABLE 1:** Baseline characteristics of male and female patients who received CAR T‐cell therapy (before and after propensity score matching).

	Before propensity score matching					After propensity score matching				
	Male		Female		*p*	Male		Female		*p*
	*N*	%	*N*	%		*N*	%	*N*	%	
No. of patients	1832	62.6%	1096	37.4%	—	1092	50.0%	1092	50.0%	—
Age, mean (SD), y	60.3 (13.7)		59.1 (13.8)		0.03	59.0 (13.7)		59.2 (13.8)		0.83
Alcohol use disorder	14	0.8%	< 10	< 10.0%	0.18	< 10	< 10.0%	< 10	< 10.0%	0.71
Anemia	16	0.9%	15	1.4%	0.21	< 10	0.6%	13	1.2%	0.18
Chronic kidney disease	201	11.0%	60	5.5%	0.00	62	5.7%	60	5.5%	0.85
Chronic liver disease	69	3.8%	49	4.5%	0.35	43	3.9%	48	4.4%	0.59
Chronic pulmonary disease	144	7.9%	117	10.7%	0.01	108	9.9%	114	10.4%	0.67
Coagulation disorder	399	21.8%	227	20.7%	0.50	218	20.0%	225	20.6%	0.71
Coronary artery disease	178	9.7%	29	2.6%	0.00	29	2.7%	29	2.7%	1.00
Congestive heart failure	58	3.2%	25	2.3%	0.16	29	2.7%	25	2.3%	0.58
Diabetes mellitus	256	14.0%	129	11.8%	0.09	118	10.8%	128	11.7%	0.50
Hyperlipidemia	503	27.5%	220	20.1%	0.00	209	19.1%	220	20.1%	0.55
Hypertension	773	42.2%	411	37.5%	0.01	390	35.7%	409	37.5%	0.40
Obesity	101	5.5%	69	6.3%	0.38	64	5.9%	68	6.2%	0.72
Obstructive sleep apnea	145	7.9%	47	4.3%	0.00	49	4.5%	47	4.3%	0.83
Peripheral arterial disease	71	3.9%	30	2.7%	0.10	29	2.7%	30	2.7%	0.89
Presence of implantable cardioverter defibrillator	< 10	< 10.0%	< 10	< 10.0%	0.25	< 10	< 10.0%	< 10	< 10.0%	1.00
Prior coronary artery bypass graft	25	1.4%	< 10	< 10.0%	0.00	< 10	< 10.0%	< 10	< 10.0%	0.56
Prior myocardial infarction	53	2.9%	12	1.1%	0.00	12	1.1%	12	1.1%	1.00
Prior percutaneous coronary intervention	65	3.5%	< 10	< 10.0%	0.00	< 10	< 10.0%	< 10	< 10.0%	0.16
Prior stroke/transient ischemic attack	44	2.4%	19	1.7%	0.23	15	1.4%	19	1.7%	0.49
Pulmonary hypertension	41	2.2%	31	2.8%	0.32	32	2.9%	30	2.7%	0.80
Substance use disorder	40	2.2%	12	1.1%	0.03	< 10	< 10.0%	12	1.1%	0.51
Smoking	549	30.0%	257	23.4%	0.00	269	24.6%	256	23.4%	0.52
Valvular heart disease	62	3.4%	28	2.6%	0.21	26	2.4%	28	2.6%	0.78

Multivariate analysis shows that female sex was not associated with worse outcomes (early mortality, adjusted odd ratios [aOR]: 1.04 [95% CI 0.69–1.57]); 30‐day readmission, aOR: 1.05 [95% CI 0.86–1.30]; nonhome discharge, aOR: 0.89 [95% CI 0.60–1.31] (Figure 1A). In terms of in‐hospital complications, female patients had higher odds of leukopenia (aOR: 1.26 [95% CI 1.06–1.50]), but lower odds of acute kidney injury (aOR: 0.68 [95% CI 0.52–0.88]). Female patients did not have higher odds of other in‐hospital complications, including infection (aOR: 1.05 [95% CI 0.88–1.26]), pulmonary embolism (aOR: 0.40 [95% CI 0.09–1.75]), thrombocytopenia (aOR: 1.01 [95% CI 0.66–1.56]), neurotoxicity (aOR: 1.06 [95% CI 0.76–1.49]), and cardiac complications (aOR: 1.40 [95% CI 0.83–2.36]) compared to the male patients (Figure 1B). In subgroup analyses for NHL, MM, and ALL (Table S1), there was no association between sex and outcomes or complications.

**FIGURE 1:**
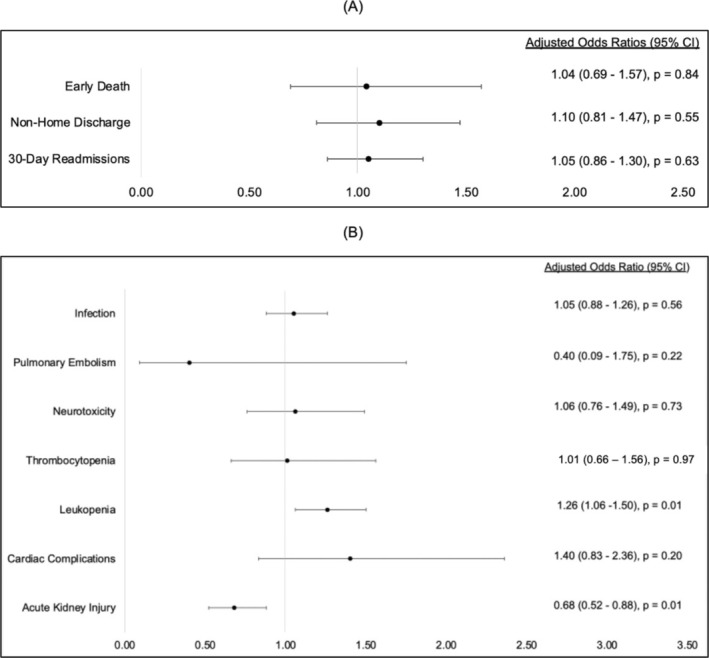
(A) Multivariate analysis of hospital outcomes using male as reference. (B) Multivariate analysis of in‐hospital complications using male as reference.

Our study identified a lower proportion of females receiving CAR T‐cell therapy, which aligns with findings from Emole et al., where females constituted only 40% of CAR T‐cell recipients [4]. This may be attributed to gender differences in meeting eligibility criteria for CAR T‐cell therapy. For instance, Tarella et al. reported a higher prevalence of primary refractory disease in B‐cell non‐Hodgkin lymphoma among males [5], and Majithia et al. noted that over 69% of relapsed multiple myeloma cases occurred in males [6]. These observations suggest gender‐related differences in disease characteristics may influence treatment access.

We found no early mortality differences between male and female patients. The common causes of mortality following CAR‐T therapy include infection, cytokine release syndrome, immune effector cell‐associated neurotoxicity syndrome (ICANS), and cardiovascular events [7, 8]. Currently, the literature on gender disparities in early mortality after CAR‐T therapy remains limited. However, a study by Buecklein et al. found that female patients had significantly better overall survival than male patients [9]. The influence of sex differences on CAR‐T therapy outcomes continues to be a topic of debate, highlighting the need for further research in this area.

Our study indicates that female patients receiving CAR‐T therapy had two times higher odds of developing leukopenia compared to their male counterparts. This is comparable to one previous study done by Xia et al. in which female patients were observed to have a higher risk of cytopenia after CAR‐T therapy [10]. It was postulated that females have stronger immune responses which result in increased inflammatory reactions and subsequent hematologic toxicity [11]. The odds of developing acute kidney injury (AKI) were 40% lower among females compared to male patients. This is consistent with a previous study that demonstrated male patients were associated with higher risks of transient AKI following CAR‐T cell therapy [12]. Males were found to be more susceptible to developing hospital‐acquired AKI compared to females [13]. This may be attributed to the protective effects of estrogen against acute kidney injury. For example, estrogen can inhibit the transforming growth factor (TGF)‐beta Type I Receptor‐Suppressor of Mothers against Decapentaplegic (SMAD) pathway, which plays a role in fibrosis and apoptosis during ischemia–reperfusion injury, ultimately contributing to kidney damage [14]. This protective role of estrogen is further supported by the increased AKI risk in diminished hormonal protection [15].

Our study is not without limitation. First, our study relies heavily on coding accuracy. Next, the out‐of‐hospital deaths that occurred before readmission were not assessed, limiting our study on early mortality. Besides, the common complications associated with CAR T‐cell therapy, including cytokine release syndrome and immune effector cell‐associated neurotoxicity syndrome, were not analyzed in our study, as their ICD‐10 codes are only available in 2021. Additionally, specific patient variables such as clinical presentation, disease stage, medications, and management are unavailable. Lastly, data with 10 or fewer observations are censored from publication in compliance with the Healthcare Cost and Utilization Project (HCUP) Data Use Agreement to ensure patient privacy and confidentiality.

Despite the limitations, our study has sufficiently demonstrated that there were no sex differences in hospital outcomes, including early mortality, 30‐day readmission, and nonhome discharge after CAR T‐cell therapy. However, females had higher odds of developing leukopenia and lower odds of acute kidney injury. These findings suggest sex‐related variations in complications, warranting further investigation.

## Author Contributions

Jia Yi Tan was involved in conceptualization, validation, visualization, project administration, and writing the original draft; Yong Hao Yeo was involved in methodology, software, data curation, investigation, validation, and formal analysis. Qi Xuan Ang was involved in methodology, software, data curation, investigation, and formal analysis. Hermon Wong Kha Kin was involved in writing the paper. Mohammad Muhsin Chisti, Daniel Ezekwudo, and Talal Hilal reviewed and edited the paper.

## Disclosure

The authors have nothing to report.

## Ethics Statement

Institutional review board review approval or informed consent is not required because the National Readmission Database is publicly available and deidentified.

## Consent

All authors gave consent for the submission of the manuscript. The population data on HCUP is de‐identified.

## Conflicts of Interest

Talal Hilal reports receiving research funding to institution from BeiGene and BMS.

## Supporting information


Table S1.

## Data Availability

The data that support the findings of this study are available from the Healthcare Cost and Utilization Project (HCUP), Agency for Healthcare Research and Quality (AHRQ). The HCUP DUA governs the disclosure and use of the data, including affirmations to protect individuals, establishments, and the database itself.
