# Supplementary material for: Sex Differences in Outcomes of Chimeric Antigen Receptor (CAR) T‐Cell Therapy
Source: Cancer Med. 2025 Mar 25;14(6):e70831. doi: 10.1002/cam4.70831 (PMC11933716; doi:10.1002/cam4.70831)
Supplement: Supplementary file 1 — Table S1. [file CAM4-14-e70831-s001.docx]

Supplemental Table 1 Subgroup Multivariate Analyses of Hospital Outcomes and Complications Using Male as Reference

| **Multivariate Analysis** | | | | |
| --- | --- | --- | --- | --- |
|  | **Adjusted Odd Ratios** | **Lower CI** | **Upper CI** | **P-value** |
| **Overall** |  |  |  |  |
| **Outcomes** |  |  |  |  |
| Early Mortality | 1.04 | 0.69 | 1.57 | 0.84 |
| 30-day Readmission | 1.05 | 0.86 | 1.30 | 0.63 |
| Non-home Discharge | 1.10 | 0.81 | 1.47 | 0.55 |
| **Complications** |  |  |  |  |
| Acute Kidney Injury | 0.68 | 0.52 | 0.88 | <0.01 |
| Cardiac Complications | 1.40 | 0.83 | 2.36 | 0.20 |
| Leukopenia | 1.26 | 1.06 | 1.50 | <0.01 |
| Thrombocytopenia | 1.01 | 0.66 | 1.56 | 0.97 |
| Neurotoxicity | 1.06 | 0.76 | 1.49 | 0.73 |
| Pulmonary Embolism | 0.40 | 0.09 | 1.75 | 0.22 |
| Infection | 1.05 | 0.88 | 1.26 | 0.56 |
|  |  |  |  |  |
| **Non-Hodgkin Lymphoma** | |  |  |  |
| **Outcomes** |  |  |  |  |
| Early Mortality | 0.98 | 0.62 | 1.57 | 0.94 |
| 30-day Readmission | 1.16 | 0.9 | 1.49 | 0.25 |
| Non-home Discharge | 1.35 | 0.96 | 1.89 | 0.09 |
| **Complications** |  |  |  |  |
| Acute Kidney Injury | 0.75 | 0.55 | 1.03 | 0.08 |
| Cardiac Complications | 1.35 | 0.74 | 2.48 | 0.33 |
| Leukopenia | 1.22 | 1 | 1.5 | 0.05 |
| Thrombocytopenia | 1.13 | 0.65 | 1.97 | 0.67 |
| Neurotoxicity | 0.95 | 0.65 | 1.38 | 0.78 |
| Pulmonary Embolism | 0.42 | 0.05 | 3.62 | 0.43 |
| Infection | 0.93 | 0.76 | 1.15 | 0.51 |
|  |  |  |  |  |
| **Multiple Myeloma** |  |  |  |  |
| **Outcomes** |  |  |  |  |
| Early Mortality | 0 | 0 | Inf | 1 |
| 30-day Readmission | 0.6 | 0.27 | 1.33 | 0.21 |
| Non-home Discharge | 0 | 0 | Inf | 1 |
| **Complications** |  |  |  |  |
| Acute Kidney Injury | 0.62 | 0.27 | 1.41 | 0.25 |
| Cardiac Complications | 0.52 | 0.09 | 2.89 | 0.46 |
| Leukopenia | 1.19 | 0.71 | 2.01 | 0.51 |
| Thrombocytopenia | 0.42 | 0.09 | 1.98 | 0.28 |
| Neurotoxicity | 1.67 | 0.35 | 7.88 | 0.52 |
| Pulmonary Embolism | 0 | 0 | Inf | 1 |
| Infection | 1.22 | 0.69 | 2.15 | 0.5 |
|  |  |  |  |  |
| **Acute Lymphocytic Leukemia** | |  |  |  |
| **Outcomes** |  |  |  |  |
| Early Mortality | 2.61 | 0.29 | 3.17 | 0.39 |
| 30-day Readmission | 0.76 | 0.22 | 2.58 | 0.66 |
| Non-home Discharge | 1.28 | 0.2 | 8.39 | 0.8 |
| **Complications** |  |  |  |  |
| Acute Kidney Injury | 1.06 | 0.23 | 4.92 | 0.94 |
| Cardiac Complications | 0 | 0 | Inf | 1 |
| Leukopenia | 0.5 | 0.18 | 1.42 | 0.19 |
| Thrombocytopenia | 0.94 | 0.09 | 9.86 | 0.96 |
| Neurotoxicity | 1.36 | 0.31 | 6.08 | 0.68 |
| Pulmonary Embolism | 0 | 0 | Inf | 1 |
| Infection | 2.16 | 0.79 | 5.92 | 0.13 |
|  |  |  |  |  |
|  |  |  |  |  |
|  |  |  |  |  |
|  |  |  |  |  |
|  |  |  |  |  |
|  |  |  |  |  |
|  |  |  |  |  |
|  |  |  |  |  |
|  |  |  |  |  |
|  |  |  |  |  |
|  |  |  |  |  |
|  |  |  |  |  |
|  |  |  |  |  |

*Inf: infinity
